# Genetic epidemiological characteristics of a Hungarian subpopulation of patients with Huntington’s disease

**DOI:** 10.1186/s12883-021-02089-9

**Published:** 2021-02-18

**Authors:** Katalin Despotov, Dénes Zádori, Gábor Veres, Katalin Jakab, Gabriella Gárdián, Eszter Tóth, Tamás Zsigmond Kincses, László Vécsei, András Ajtay, Dániel Bereczki, Péter Klivényi

**Affiliations:** 1 Department of Neurology, University of Szeged, 6 Semmelweis Street, Szeged, 6725 Hungary; 2 Department of Neurology, Semmelweis University, Budapest, Hungary; 3 MTA-SE Neuroepidemiological Research Group, Budapest, Hungary

**Keywords:** Demographics, Huntington’s disease, Population genetics, Trinucleotide repeat expansion

## Abstract

**Background:**

Recent advances in therapeutic options may prevent deterioration related to Huntington’s disease (HD), even at the pre-symptomatic stage. Be that as it may, a well-characterized patient population is essential for screening and monitoring outcome. Accordingly, the aim of this study was to describe the characteristics of a Hungarian subpopulation of HD patients and mutation carriers diagnosed at the University of Szeged.

**Methods:**

We conducted a search for International Classification of Diseases (ICD) code G10H0 in the local medical database for the period of 1 January 1998 to 31 December 2018.

**Results:**

We identified 90 HD cases (male: 45, female: 45) and 34 asymptomatic carriers (male: 15, female: 19). The median age of onset was 45 years (range: 16–79). There were 3 cases of juvenile onset (3.3%), and 7 of late disease onset (7.8%). The median repeat length was 43 (range: 36–70) for the pathological and 19 for the non-pathological alleles (range: 9–35). 17.5% of the pathological alleles were in the decreased penetrance range, while 7% of non-pathological alleles were intermediate.

**Conclusions:**

The genetic and clinical features of the population examined in the present study were in line with the previous Hungarian study, as well as with international literature. The exceptions were the higher ratio of reduced penetrance and intermediate alleles.

**Supplementary Information:**

The online version contains supplementary material available at 10.1186/s12883-021-02089-9.

## Background

Huntington’s disease (HD) is a hereditary neurodegenerative disorder characterised by choreiform movements, cognitive dysfunction, behaviour and mood problems [1–4]. The worldwide prevalence of HD is estimated to be 2.71/100000, with a significantly higher prevalence (5.7/100000) and incidence (0.11–0.8/100000) in Europe and North America compared to Asian populations (prevalence: 0.4/100000, incidence: 0.046–0.16/100000) [2, 5].

HD is caused by a CAG trinucleotide repeat expansion in the first exon of the *HTT* gene, located on chromosome 4p16.3, showing an autosomal dominant pattern of inheritance [3, 6–10]. Repeat length of 26 or less is considered normal [3, 8, 11]. The repeats falling between 27 and 35 are referred to as intermediate alleles (or large normal alleles), which are not considered to be pathogenic, but, due to meiotic instability, they are prone to expansion, often leading to the development of HD in the next generation [3, 11, 12]. In some cases, however, the presence of these alleles has been associated with subtle HD-like symptoms [11, 13]*.* If the expansion exceeds 35 repeats, it becomes pathogenic, with the range 36–39 showing decreased penetrance, and a length of 40 or longer showing full penetrance [3, 4, 8]. The age of onset and the CAG repeat length of the pathological allele shows a strong inverse correlation, though it is widely debated in the literature whether repeat length by itself can be used to accurately predict disease onset and duration [3, 14]. Several studies suggest that the age of onset can be influenced by other genetic factors, including the length of the non-pathological allele [3, 15]*.* HD is characterised by genetic anticipation, the phenomenon that each successive generation demonstrates an earlier age of onset. It is based on the meiotic instability of the mutation, and is usually associated with paternal transmission [3, 8].

The age of disease onset varies widely, the average being estimated between 30 and 50 years [1, 3, 4, 6, 7], but it can occur during early childhood as well as over the age of 60 [3, 4, 8]. Cases where the disease manifests itself before the age of 20 are called juvenile type HD, whereas cases presenting symptoms after the age of 60 (in some papers, after the age of 50), are termed late onset HD [8, 9, 16–19]. Juvenile HD accounts for approximately 5% of all HD cases, and are usually characterised by large repeat numbers and paternal transmission [19, 20]. Some of these patients present atypical symptoms like rigidity, brady-hypokinesis, postural instability and dysarthria, a phenotype referred to as the Westphal-variant [21]. The frequency of late-onset HD varies widely in different studies with a range between 4.4–25% [16, 22]. In these cases, the course of the disease tends to be mild, and repeat lengths are usually below 50 [16, 22]*.* Regarding disease course, HD is a progressive condition, resulting in death within 10–20 years after the first appearance of symptoms [9]. Juvenile onset is associated with shorter and late onset with longer disease duration [3, 20].

The aim of the current study is to evaluate the characteristics of genetic and clinical features of a Hungarian subpopulation of HD patients and asymptomatic carriers, and to compare the obtained results with those of a previously published study from the same university in 1999 [10], as well as with available international literature data.

## Methods

We performed a search for International Classification of Diseases (ICD) code G10H0 in the electronic medical database of the Department of Neurology, University of Szeged, Hungary, for the period between 1 January 1998, and 31 December 2018. We also reviewed the paper-based medical archives of our out-patient unit for that same time interval. Case assessment was conducted by reviewing medical records of patient history and genetic findings. HD was defined by the presence of a pathological mutation in the *HTT* gene (CAG repeat length of 36 or above) and related unequivocal neurological and/or psychiatric symptoms. A mutation carrier was defined as having a pathological allele but no evidence of clinical symptoms. Those who were first diagnosed as mutation carriers and developed symptoms at a later time were placed in the HD patient group. Age of onset was established by the first appearance of neurologic or psychiatric symptoms which could be directly associated with HD. Time of diagnosis was defined as the earliest available date in the records when both clinical symptoms and confirmatory genetic results were present, or, when not available, the first reference in the records as a genetically defined case. Regarding paternal or maternal inheritance, the presence of HD in the affected ancestors was based on clinical data. To put the number of newly diagnosed HD cases into context, the total number of patients seen in the in- and outpatient units of our department was counted for each year in the period of interest using the electronic medical database of our department. We also assembled a control group (*n* = 62, male: 32, female: 30), which, as illustrated in Additional file 1, consisted of unaffected, asymptomatic relatives of HD patients who were tested at their own request in order to determine potential carrier status, and individuals, referred to our department for diagnostic purposes, who were suspected of suffering from HD based on clinical presentation, but tested negative for a pathological expansion in the *HTT* gene.

All statistical calculations were performed with the use of the freely available R software (R Development Core Team). We applied multiple linear regression models to find out if there was a difference or interaction between the different variables.

## Results

Based on medical records from 1 January 1998 to 31 December 2018, we identified 90 cases of HD (male: 45, female: 45) and 34 asymptomatic carriers (male: 15, female: 19). In the HD patient group, there were a total of 6 individuals – 3 male, 3 female – who were first diagnosed as mutation carriers and later developed symptoms. The median number of newly diagnosed HD patients per year was 3, with a range of 1–8 new cases/year, accounting for an average of 0.025% of all patients seen in our department each year (range: 0.005–0.079%). The core clinical features of the disease were present in all individuals in the HD patient group. The medians and ranges for age of onset are indicated in Table 1/A, and a comparison of juvenile and late onset cases examined in this study is presented in Table 1/B.

**Table 1:** Age of onset in HD (A) and comparison of juvenile and late onset (B)

A)	All	Male	Female
Available (n)	79	40	39
Median (years)	45	43.5	45
Range (years)	16–79	18–79	16–78
B)	Juvenile onset	Late onset	
Number of cases (male/female)	3 (2/1)	7 (1/6)	
Percent of all cases	3.3%	7.8%	
Age of onset	16, 18, 19 years	61, 61, 62, 62, 71, 78, 79 years	
CAG repeat length	70, 52, 59 repeats, respectively	45, 45, 40, 37, 39, 37, 40 repeats, respectively	
Affected parent known	3 cases	2 cases	
Parental transmission	Paternal (3/3)	Maternal (2/2)	

One male patient with juvenile HD had a repeat length of 52. He exhibited behavioural problems and learning difficulties in early childhood and motor onset at the age of 18. A female patient, with a repeat length of 70, presented atypical symptoms such as rigidity, cognitive dysfunction, hypokinesis, postural instability, dysarthria and dysphagia at the age of 16.

The first presenting signs of HD patients were determined from available documentation in 75 cases. The distribution of these symptoms is indicated in Table 2.

**Table 2:** Distribution of first presenting symptoms in the HD patient group (available in 75 cases)

First symptom	Motor = n (%)	Cognitive = n (%)	Psychiatric = n (%)	Total = n (%)
Motor = n (%)	42 (46.7%)	17 (18.9%)	3 (3.3%)	62 (68.9%)
Cognitive = n (%)	17 (18.9%)	2 (2.2%)	0 (0%)	19 (21.1%)
Psychiatric = n (%)	3 (3.3%)	0 (0%)	11 (12.2%)	14 (15.6%)
Total = n (%)	62 (68.9%)	19 (21.1%)	14 (15.6%)	75 (100%)

**Fig. 1:**
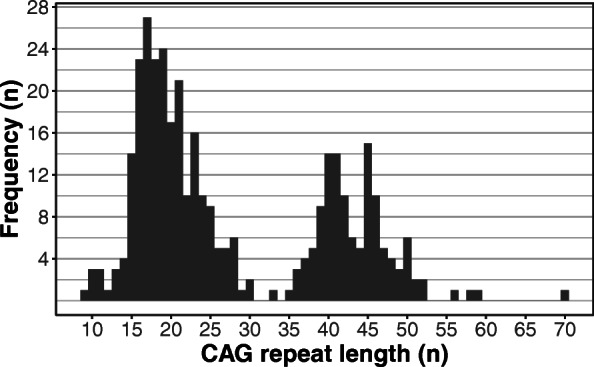
Allele frequencies of HD (*n* = 86), carrier (*n* = 34) and control groups (*n* = 62). As indicated, the majority of the pathological alleles fall between 36 and 50 repeats, and those longer than 55 are very rare. Regarding the non-pathological range, the most frequent alleles are between 15 and 25 repeats, while intermediate alleles, especially those between 30 and 35 are also rare

The median time between disease onset and the date of diagnosis was 3.5 years (range: 0–20 years). The frequencies of all alleles in HD, carrier and control groups are presented in Fig. 1.

Exact CAG repeat lengths of pathological alleles were available in 86 HD cases (male: 42, female: 44) and in 34 carriers, whereas the sizes of normal alleles were known in 75 HD patients and 31 carriers. The parameters (median, range) and distribution of pathological and non-pathological alleles in each group are described in Table 3. As indicated in Table 3, regarding alleles in the non-expanded range, there is no statistical difference between HD, carrier and control groups.

**Table 3:** Distribution of CAG repeat lengths in pathological and non-pathological ranges by group and gender

	HD group	Carrier group	Control group (Alleles [individuals])	All pathological alleles	All non-pathological alleles
CAG repeats of pathological range (≥ 36)					
N (male/female)	86 (42/44)	34 (15/19)	–	120 (57/63)	–
Median (male/female)	45 (45/43.5)	41 (42/41)	–	43 (42/44)	–
Range (male/female)	36–70 (38–59/36–70)	36–52 (36–52/36–49)	–	36–70 (36–59/36/70)	–
CAG repeats of decreased penetrance (36–39)					
N (male/female)	12 (3/9)	9 (2/7)	–	21 (5/16)	–
Percent of respective group members	13.3%	26.5%	–	17.5%	–
CAG repeats of non-pathological range (< 36)					
N (male/female)^a^	75 (34/41)	30 (13/17)	124 (64/60)	–	229 (111/118)
Median (male/female)	18 (18/19)	19 (19/19)	[62 (32/30)]	–	19 (19/19)
Range (male/female)	9–28 (10–28/9–27)	14–24 (16–23/14–24)	20 (20/19) 10–35 (10–33/11/35)	–	9–35 (10–33/9–35)
CAG repeats of intermediate range (27–35)					
N (male, female)	3 (2/1)	0	13 (7/6)	–	16 (9/7)
Percent of respective group members	4%	0	10.5% [21%]	–	7%

^a^The repeat number of the normal allele was not available in all HD cases and carriers

**Fig. 2:**
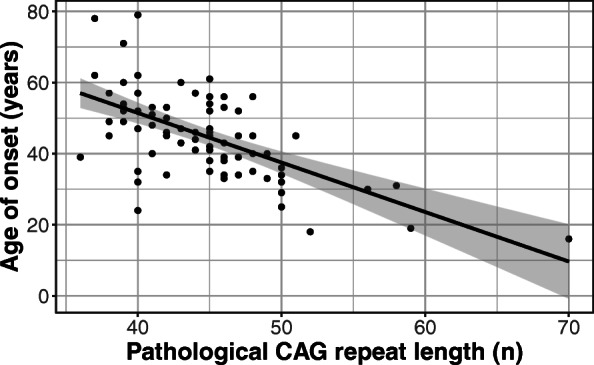
Correlation between the age of onset and pathological CAG repeat length in HD patients. An inverse correlation is presented in the figure, indicating that higher repeat lengths are associated with an earlier age of onset. It is also shown that the data are more scattered along the lower range of the spectrum and less so in the higher range, although the small number of cases with high repeat numbers (55<) poses a limitation to such conclusions. The gray zone represents the 95% confidence interval

The age of onset showed a strong inverse correlation with the pathological CAG repeat length (F_(1, 76)_ = 47.37; R^2^ = 0.38; *p* < 0.001), whereas no statistically significant correlation was found between the age of onset and the size of non-expanded alleles (F_(1, 69)_ = 1.94; R^2^ = 0.013; *p* = 0.17). When we investigated these alleles in the same model, the results did not change (F_(3, 67)_ = 16.86; R^2^ = 0.41; *p* < 0.001), and there was no interaction between the allele variables (*p* = 0.17; Fig. 2).

Alleles of decreased penetrance were identified in 9 carriers (26.5%, male: 2, female: 7) and 12 HD cases (13.3%, male: 3, female: 9), representing 17.5% of pathological alleles. In this group of HD patients, the mean age of onset was 53.5 years (range: 39–78 years)*.* Out of these 21 individuals, an affected parent was known in 11 cases (mother: 5, father: 6).

Basic genetic and clinical information on symptomatic control group individuals with two normal alleles is presented in Additional file 2.

We identified 16 individuals – 3 in the HD group and 13 in the control group – who carried intermediate alleles. These intermediate alleles represented approximately 10.5% of control group alleles, 7% of all non-pathological and 4.6% of all alleles assessed in this study. The 13 individuals carrying intermediate alleles (male: 7, female: 6) represented 21% of all controls. No individuals with 2 intermediate alleles were found. Eight of these controls, all of whom (as indicated in Additional file 1), were referred to our department for diagnostic purposes, presented with HD-like symptoms, such as chorea, perioral dyskinesis, and cognitive decline (5 males with repeat lengths of 27, 28, 28, 30, 33, and 3 females with repeat lengths of 28, 28, 35, respectively). Three of them had additional symptoms, such as ataxia, dysphagia, dysarthria and convulsions. One person showed atypical extrapyramidal signs (myoclonus, cervical dystonia). Evidence for alternative diagnosis (alcohol and medication abuse) was provided only in one of these cases. Only one individual had a positive family history: a woman with alleles of 15 and 35 repeat lengths, who presented with minor signs of movement disorder showing no progression during follow-up examinations. Her mother was diagnosed with HD (there is no evidence of genetic confirmation) and she had several other relatives with similar symptoms.

**Fig. 3:**
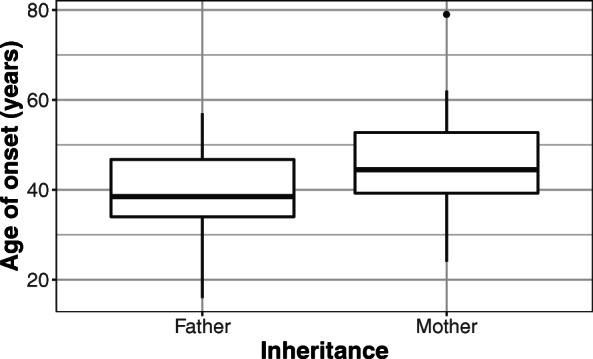
Comparison of paternal (*n* = 25) and maternal (*n* = 27) inheritance in term of age of onset. Though the difference is not statistically significant, the figure illustrates that paternal transmission tends to be associated with an earlier age of onset. The data are presented as median, IQR, minimum-maximum

**Fig. 4:**
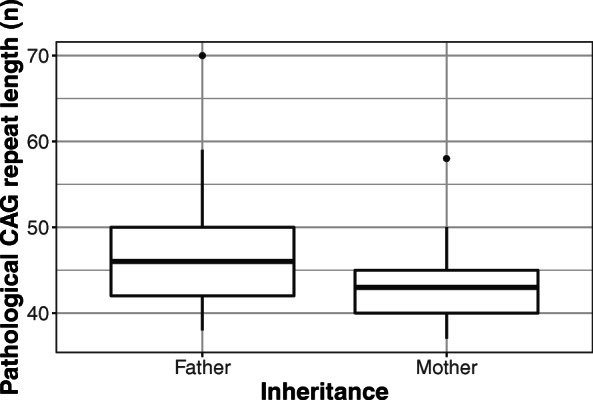
Comparison of paternal (*n* = 25) and maternal (*n* = 27) inheritance in term of pathological CAG repeat length. Though the difference is not statistically significant, the figure illustrates a tendency toward slightly higher repeat lengths in cases of paternal transmission. The data are presented as median, IQR, minimum-maximum

Fifty-two HD patients were found to have a positive family history. There were 25 patients with paternal and 27 with maternal inheritance. Regarding the carrier group, evidence of an affected parent was found in 26 (14 with maternal and 12 with paternal inheritance) out of 34 cases. Though the median age of onset was lower in cases of paternal (38.5, range: 16–57 years) than in that of maternal transmission (44.5, range: 24–79 years), and the median repeat length was higher in cases of paternal (46, range: 38–70) compared to maternal inheritance (43, range: 37–58), the difference was not statistically significant (*p* = 0.0612 for age of onset and *p* = 0.1 for pathological allele size) (Figs. 3 and 4).

## Discussion

In recent years, there have been major advances in molecular therapeutic options for HD. As a prelude to joining international clinical trials, the detailed characterization of target patient populations is essential, enabling clinicians to predict and better understand differences in therapeutic response [23–25].

Accordingly, in this retrospective study, we aimed to characterise 90 HD cases (male: 45, female: 45), and 34 carriers.

The median age of onset (45 years) was close to the European Huntington’s Disease Network’s data for Central Europe (47 years) but it was lower than those of Northern (50 years) or Southern Europe (49 years) as well as that of the United Kingdom (49 years) [4, 6, 9].

The ratio of juvenile onset cases (3.3%) was slightly lower than the 5% described in other studies [16, 22]. All of these patients had a repeat length above 50 and a positive family history where the affected parent was the father. One patient with juvenile onset presented with the atypical Westphal phenotype [21]. Seven patients with late disease onset were identified, representing 7.8% of all cases, which is well within the range (4.4–25%) previously reported [16, 22]. Each of these patients had a repeat length below 46, and evidence of an affected parent was present in only 2 out of 7 cases.

The ratio of pure motor onset (47.6%) was similar to the data of the European Huntington’s Disease Network (48%), while psychiatric (12.2%) and cognitive (2.2%) onset was less frequent than reported in the same study (19.6 and 8.4%, respectively). This difference might be partly explained by the higher ratio of mixed onset cases (22.2%) in the present study compared to the report of the European Huntington’s Disease Network (13.2%) [6].

Compared to the previous study from Hungary (median: 43, range: 37–70) and the international literature (median: 42–44, range: 36–121), we found that the pathological repeat lengths of HD patients and asymptomatic carriers was similar [6, 7, 10, 26, 27]*.* Alleles with decreased penetrance were found in 9 carriers (26.5%) and 12 HD patients (13.3%), representing approximately 17.5% of all pathological alleles found in this study, which is higher than the data reported by the European Huntington’s Disease Network (3.1% for all participants, 1.8% for Central, 10% for Northern and 2.2% for Southern Europe) [6]. As expected, the median age of onset (53.5 years) of patients in this range was higher than that of the whole HD group (45 years) [12, 28]*.* The frequency of intermediate alleles, either in the control group alleles (10.5%) or in all non-expanded alleles from the three groups (7%), was higher than those reported from most populations (0.45–6%), the exceptions being the findings of 2 Brazilian cohorts with frequencies of 7–8.7% [11, 13, 23, 29, 30]. There are several reports associating intermediate alleles to HD-like clinical and pathological findings, thus the assessment of these alleles is gaining increased attention despite the still controversial data [11, 12, 30]*.* In the current study, we identified 8 individuals out of 13 controls with intermediate alleles presenting symptoms similar to that of HD, although lacking further clinical data or pathological confirmation, it is unclear whether there is a causative relationship between these alleles and the presenting symptoms.

The median length of non-expanded CAG repeat alleles (19) was similar to that found in the previous Hungarian study (18) and those reported from other Caucasian populations (17.1–19.3), but higher than in Asian or African ethnic groups (16.2–17.7), [10, 23]*.*

The strong inverse correlation between age of onset and pathological repeat size was established in the current study as well (R^2^ = 0.38; *p* < 0.001). However, there was no significant association between the age of onset and the length of the non-pathological allele. It has been demonstrated that the expanded allele explains about 66–67.3% of the variance in age of onset, whereas the effect of the non-expanded allele is relatively small (approximately 1%) [3, 15]. Furthermore, it was proposed that the effect of the normal allele becomes evident among individuals with large pathological repeat lengths [15], the population of which accounts for only a small proportion of patients in our study (25.6% with repeat sizes above 46 and only 8% exceeding 50).

Although the lack of detailed clinical characterization is a limitation of the study, the symptoms of HD, especially at an early stage, are rather diverse and aspecific, therefore, a diagnosis of HD can only be established in possession of correlating genetic evidence.

In conclusion, the genetic and clinical features of the populations examined in the present study were in accordance with the previous Hungarian study as well as with international literature data, except for the higher frequency of intermediate alleles and individuals with reduced penetrance alleles. The presence of these alleles is gaining importance in light of increasing evidence of disease modifying genetic factors, such as the loss of interruption variants, which have been extensively investigated in the past 2 years [31, 32], owing to developments in analytic technologies. They are considered to cause CAG repeat length underestimation with the currently, most widely, used diagnostic methods. Additionally, some authors suggest that these variants not only influence the age of onset, but, in individuals carrying reduced penetrance alleles, they might play a major role in the manifestation of the disease [31, 32]. These factors have not yet been thoroughly analysed in individuals carrying intermediate alleles and presenting neurological symptoms, which could serve as a target for future studies.

## Supplementary Information

**Additional file 1.** Composition of the control group. This flowchart illustrates the individuals with CAG repeat lengths in the non-pathological range (< 36), highlighting those carrying intermediate alleles.

**Additional file 2.** Symptomatic control group individuals with biallelic wild-type genotypes. This table describes the clinical presentation of individuals tested for differential diagnostic purposes and were found to carry alleles with repeat lengths < 27.

## Data Availability

The datasets used and/or analysed during the current study are available from the corresponding author on reasonable request.

## Abbreviations

HD: Huntington’s Disease
ICD: International Classification of Diseases
