# Supplementary material for: Genetic epidemiological characteristics of a Hungarian subpopulation of patients with Huntington’s disease
Source: BMC Neurol. 2021 Feb 18;21:79. doi: 10.1186/s12883-021-02089-9 (PMC7890867; doi:10.1186/s12883-021-02089-9)
Supplement: Supplementary file 1 — Additional file 1. Composition of the control group. This flowchart illustrates the individuals with CAG repeat lengths in the non-pathological range (< 36), highlighting those carrying intermediate alleles. [file 12883_2021_2089_MOESM1_ESM.pdf]

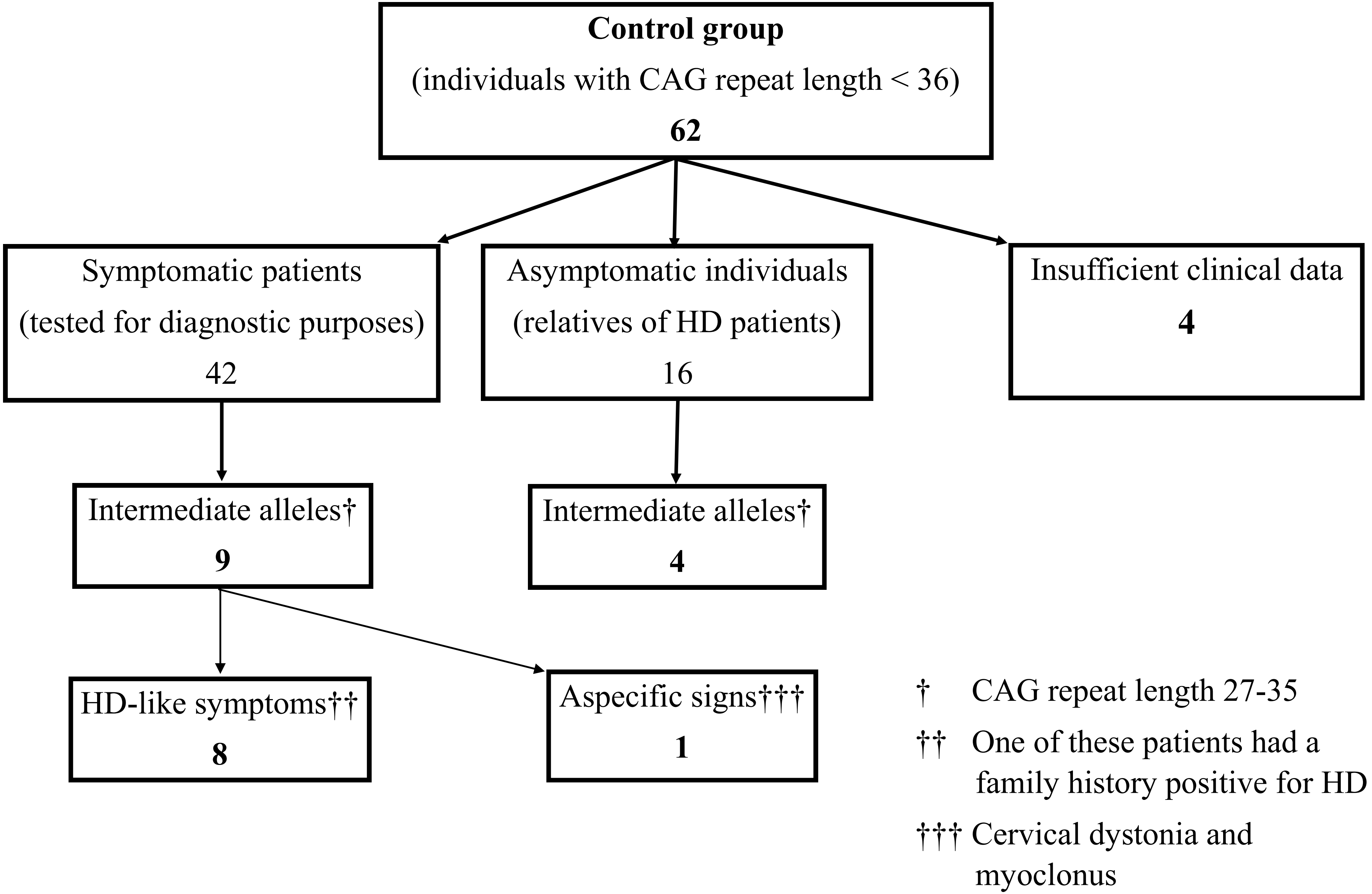
Control group
(individuals with CAG repeat length < 36)
62
Symptomatic patients
(tested for diagnostic purposes)
42
Asymptomatic individuals
(relatives of HD patients)
16
Insufficient clinical data
4
Intermediate alleles†
9
Intermediate alleles†
4
HD-like symptoms††
8
Aspecific signs†††
1
† CAG repeat length 27-35
†† One of these patients had a family history positive for HD
††† Cervical dystonia and myoclonus
