# Supplementary material for: Genetic epidemiological characteristics of a Hungarian subpopulation of patients with Huntington’s disease
Source: BMC Neurol. 2021 Feb 18;21:79. doi: 10.1186/s12883-021-02089-9 (PMC7890867; doi:10.1186/s12883-021-02089-9)
Supplement: Supplementary file 2 — Additional file 2. Symptomatic control group individuals with biallelic wild-type genotypes. This table describes the clinical presentation of individuals tested for differential diagnostic purposes and were found to carry alleles with repeat lengths < 27. [file 12883_2021_2089_MOESM2_ESM.pdf]

Additional file 2. Symptomatic control group patients with biallelic wild-type (WT) genotype

**Code**	**Gender†**	**Genotype (CAG repeat number)**	**Neurologic symptoms**	**Cognitive/psychiatric symptoms**
WT01	2	22/22	4 limbs chorea, apraxia	dementia, hallucinations
WT02	1	23/23	4 limbs hyperkinesis, ataxia	dementia
WT03	2	19/15		dementia
WT04	1	17/16	generalized chorea, dystonia, facial dyskinesis	
WT05	2	25/19	chorea, dystonia	
WT06	2	25/16	chorea, ataxia	paranoid disorder
WT07	1	26/20	right limbs tremor	Dementia
WT08	2	20/18	tremor, rigidity, brady-hypokinesis, cervical dystonia, hypomimia, dysprosodia	cognitive dysfunction, psychosis
WT09	2	17/17	generalized chorea, ataxia, myoclonus, dysarthria, gaze palsy	
WT10	2	22/25	left limb chorea, facial dyskinesis, left limbs weakness	
WT11	1	15/21	4 limb hyperkinesis, facial dyskinesis	
WT12	1	18/18	facial dyskinesis, cervical dystonia	psychosis, depression
WT13	1	21/21	facial dyskinesis, tremor, rigidity	Dementia
WT14	1	18/16	dystonia, tremor, rigidity, brady-hypokinesis, postural instability, hypomimia, hypophonia	
WT15	1	20/25	4 limbs chorea, facial dyskinesis, cervical dystonia, hypomimia	
WT16	2	21/19	4 limbs hyperkinesis, dystonia, ataxia, tremor, hypomimia	
WT17	2	25/16	dystonia, ataxia, gaze palsy	
WT18	1	23/23	dystonia, tremor, myoclonus	depression
WT19	1	23/16	upper limbs hyperkinesis, facial dyskinesis, ataxia	psychosis, bipolar disorder
WT20	2	19/11	generalized hyperkinesis	psychosis, depression
WT21	2	19/24	4 limbs chorea, left upper limb dystonia	
WT22	2	18/11	4 limbs chorea, ataxia, dysarthria	dementia, emotional lability
WT23	2	12/16	Chorea	
WT24	2	21/20	4 limbs choreo-athetosis, blepharospasm, ataxia, dysarthria, dysphagia	Dementia

WT25	2	17/19	chorea, ataxia, tremor	dementia, mood disorder
WT26	1	15/15	ataxia, dysarthria	Dementia
WT27	2	24/24	generalized chorea	
WT28	2	13/13	cervical dystonia	psychosis, bipolar disorder
WT29	2	23/23	4 limbs chorea-ballism, facial dyskinesis, dysarthria	dementia, depression
WT30	2	25/16	Dyskinesis	paranoid disorder
WT31	2	22/11	left limbs chorea, dysarthria	
WT32	1	15/24	right lower limb chorea, bradykinesis	Dementia
WT33	1	17/17	generalized chorea	

†Gender: 1 = male, 2 = female
